# Addressing language challenges in bilingual neuropsychological assessments at the South Texas Alzheimer's Disease Research Center (ADRC)

**DOI:** 10.1002/alz.70800

**Published:** 2025-10-16

**Authors:** Stephanie Santiago‐Mejias, Gabrielle Hromas, Ashley LaRoche, David A. Gonzalez, Robin C. Hilsabeck, Katya Rascovsky, Silvia Mejia Arango, Amy Werry‐McFarlin, Claudia L. Satizabal, Hector Trevino, Monica Goss, Jennifer Del Bosque, Amaya Seidl, Roberto Garcia, Marialy Salinas Valdez, Angel Velarde, Jessica Zapata, Samantha Gates, Patricia Hernandez, Juan Toranzo, Marucela Uscamayta Ayvar, Denisse Garcia Cisneros, Vanessa M. Young, Sudha Seshadri, Anna Campbell Sullivan

**Affiliations:** ^1^ Glenn Biggs Institute for Alzheimer's and Neurodegenerative Diseases University of Texas Health Science Center at San Antonio San Antonio Texas USA; ^2^ Department of Neurological Sciences Rush University Chicago Illinois USA; ^3^ Department of Neurology Perelman School of Medicine University of Pennsylvania Philadelphia Pennsylvania USA; ^4^ Institute of Neuroscience School of Medicine University of Texas Rio Grande Valley Harlingen Texas USA

**Keywords:** Alzheimer's disease, bilingualism, cognitive testing, dementia, language dominance, neuropsychological assessment

## Abstract

**INTRODUCTION:**

Neuropsychological assessment of bilingual (English/Spanish) individuals presents challenges that can impact test validity. Language proficiency influences cognitive performance, yet clear guidelines for determining the appropriate test language are lacking. We describe our experiences at the South Texas Alzheimer's Disease Research Center (STAC) in addressing these challenges within the context of National Alzheimer's Coordinating Center (NACC) Uniform Data Set (UDS) assessments and broader Alzheimer's Disease Research Center (ADRC) protocols. We outline steps toward a structured language assessment approach.

**METHODS:**

We implemented a process to assess language proficiency, integrating self‐reported and objective measures, including the language dominance index (LDI). Case examples illustrate the impact of language on cognitive testing.

**RESULTS:**

Challenges included discrepancies between self‐reported and objective language proficiency, language switching during assessments, and resistance to testing in the dominant language.

**DISCUSSION:**

Language assessment improves test validity and research consistency. Future efforts should refine bilingual assessment methods and establish standardized protocols.

**Highlights:**

- Systematic test language selection may improve accuracy in bilingual assessments.
- Discrepancies in reported versus objective language proficiency challenge bilingual assessments.
- Language evaluation guidelines are needed to improve test validity and data consistency.

## 1 BACKGROUND

The Hispanic/Latine population in the United States is projected to reach 111 million by 2060,^1^, ^2^ and older Hispanic/Latine adults are 1.5 times more likely to develop dementia than Non‐Hispanic/Latine White adults.^3^ Nevertheless, they remain underrepresented in Alzheimer's disease and related disorders (ADRD) research.^4^ Barriers include limited culturally appropriate resources, insufficient culturally and linguistically representative staffing, and study procedures not tailored to these communities.^5^ In response, the National Institute on Aging (NIA) has funded new Alzheimer's Disease Research Centers (ADRCs).^6^ The South Texas Alzheimer's Disease Research Center (STAC) was established in 2021. Operating across three sites—University of Texas (UT) Health San Antonio, the University of Texas Rio Grande Valley, and the UT Education and Research Center at Laredo—STAC currently follows 626 active participants, 53% of whom identify as Hispanic/Latine, all with varying levels of English/Spanish bilingualism, of whom 66 were analyzed.

The Uniform Data Set (UDS) introduced the Spanish‐translated neuropsychological battery in 2007.^7^ In 2017, the CLS: Linguistic History Form was implemented to assess language preferences and proficiency.^8^ Despite these advancements, challenges remain in standardizing UDS data collection and interpretation across sites. Linguistic factors encompass not only structural aspects of language, but also the everyday social context of language use, such as language of schooling^9^ and frequency of exposure,^10^ which together influence lexical access and shape test performance. These factors influence how participants engage with assessment materials,^11^ and language proficiency differences can create inconsistencies in comprehension and response patterns, affecting data accuracy.^12^ In this article, we examine the common obstacles we have encountered when evaluating bilingual (English/Spanish) participants, focusing on language‐related issues. We outline the steps we are taking to develop a structured procedure for assessing language proficiency to optimize neuropsychological testing and present case examples that inform the development of practical guidelines for working with bilingual individuals.

### 1.1 Neurocognitive considerations

Bilingual individuals use each language less frequently than monolinguals, which can reduce vocabulary in both languages.^13^, ^14^ According to the “weaker links” hypothesis, this lower use weakens semantics‐phonological connections,^15^ affecting retrieval of low‐frequency words, especially on tasks such as picture naming.^16^ This effect is more pronounced when schooling and everyday communication occur in the other language,^9^ thereby limiting exposure to the low‐frequency words being tested. In addition, bilingual individuals often switch between languages,^17^ as both remain active to some degree, even when only one is being used.^18^ Research shows that bilingualism may provide neurocognitive benefits, such as improved executive function, coding, and processing speed tasks,^19^ and may protect against mild cognitive impairment (MCI) and dementia.^20^, ^21^

Neuroimaging studies suggest that bilingualism alters brain structure and organization,^22^ particularly in regions associated with executive control and language (e.g., inferior parietal lobule, anterior cingulate cortex, and left caudate nucleus).^23^, ^24^, ^25^, ^26^, ^27^ The first acquired language (L1) and L2 share brain regions involved in syntax processing (e.g., left inferior frontal gyrus and precentral gyrus).^23^ However, complex tasks in L2 show greater activation, suggesting increased cognitive load during L2 processing.^23^, ^28^, ^29^ These adaptations reflect the increased cognitive demands of managing two languages and emphasize the need to account for bilingual status when interpreting cognitive test results.

Bilingual proficiency varies by age, education, cultural background, and language exposure.^30^, ^31^, ^32^ Conversational fluency differs from cognitive academic language proficiency (CALP), which requires complex reasoning.^33^ Individuals who appear fluent in casual conversation may not always be proficient in using that language for academic or cognitive tasks.^34^, ^35^

### 1.2 Evaluation considerations

Typically, neuropsychological evaluations rely on self‐reported language preference,^36^, ^37^, ^38^ which may be biased by societal pressures. For example, in contexts where English is seen as a marker of social standing, bilingual individuals might choose English over their native language for assessments, despite lower proficiency, to avoid perceived discrimination.^39^, ^40^ Clinicians and researchers have been encouraged to utilize both self‐reported and objective measures of language proficiency.^41^, ^42^ Although the CLS: Linguistic History Form documents linguistic background,^43^ it remains inherently subjective and can be influenced by external factors and individual bias. The language dominance index (LDI), developed by Suarez and colleagues,^44^ offers an objective method for examining how proficiency in second language (e.g., English proficiency) might influence performance on native language assessments (e.g., the performance of Spanish‐speaking individuals on Spanish assessments). Integrating objective measures with self‐report data may help achieve a more accurate and unbiased measurement of language proficiency, enhancing assessment sensitivity, as even highly proficient English‐speaking Hispanic/Latine individuals may perform differently from their non‐Hispanic/Latine counterparts due to the nuances of bilingualism.^45^, ^46^

## 2 METHODS

All procedures were approved by the UT Health San Antonio Institutional Review Board (IRB # 20210026HU), participants provided written informed consent, and all data have been de‐identified or generalized to prevent re‐identification.

### 2.1 STAC Hispanic/Latine participants who completed the LDI and CLS

As of December 2024, 66 Hispanic/Latine participants at the STAC had completed the LDI and CLS. The sample was well educated (mean 15 years of education; median 14 [range 9–20]) and predominantly female (59%) (Table 1). To better understand language proficiency in this highly diverse sample, the LDI was implemented in 2023 as an objective measure to assess language dominance. Participants included in the present analysis were drawn from the larger cohort based on self‐reported English–Spanish bilingualism following this implementation. Participants enrolled prior to implementation, those not identifying as bilingual, or those with incomplete LDI and CLS data were excluded.

**TABLE 1 alz70800-tbl-0001:** Demographics and language characteristics of Hispanic/Latine participants who completed LDI and CLS Linguistic History Form at the STAC (ss of December 2024).

Characteristic (at first visit)	Overall (*n* = 66)	English testing (Visit 1) (*n* = 59)	Spanish testing (Visit1) (*n* = 7)
Age, mean (SD)	73.63 (9.2)	74.16 (8.8)	69.14 (11.7)
Female, *n* (%)	39 (59.1)	34 (57.6)	5 (71.4)
Education (years), mean (SD), median [min–max]	14.65 (2.8); 14 [9–20]	14.59 (2.7); 14 [9–20]	15.14 (3.9); 15 [9–20]
Self‐reported preferred language, *n* (%)	62 (94) English 4 (6) Spanish	–	–
Self‐reported preferred language (Δ)	–	3	1
CLS: Linguistic History Form			
Years in English environment, mean (SD)	63.97 (20.9)	65.39 (20.6)	52.00 (21.0)
Years in Spanish environment, mean (SD)	8.41 (17.8)	6.80 (15.8)	22.00 (27.9)
% Daily English use, mean (SD)	69.12 (27.6)	72.73 (25.1)	38.71 (31.7)
% Daily Spanish use, mean (SD)	30.88 (27.6)	27.27 (25.1)	61.29 (31.7)
CLS Average English level mean (SD)	6.5 (0.7)	6.5 (0.6)	5.8 (1.2)
CLS Average Spanish level mean (SD)	5.1 (1.3)	4.9 (1.3)	6.2 (1.0)
CLS score	0.43 (0.07)	0.42 (0.06)	0.52 (0.05)
LDI dominance, mean (SD)	0.56 (0.08)	0.58 (0.07)	0.45 (0.07)

*Note*: Demographic and language characteristics of Hispanic/Latine participants from the STAC who completed the LDI and the CLS: Language History Form, based on data collected through December 2024.

Abbreviations: Δ, change; LDI, language dominance index; SD, standard deviation

### 2.2 Neuropsychological assessment of English/Spanish bilingual individuals at the STAC

During the initial recruitment phone call, research staff directly ask prospective participants whether they use both English and Spanish (e.g., “Do you speak both English and Spanish?” / “Would you consider yourself bilingual?”) and, if yes, they record their preferred language for communication. When a participant reports any degree of bilingualism, the research staff follows standard procedures to assess language proficiency and guide test administration. Bilingual examiners are assigned to visits when recruitment indicates that a participant may be bilingual. Although the ADRC team was predominantly monolingual from 2021 to 2023, a cross‐training initiative launched in 2023 expanded our bilingual workforce, and a bilingual examiner is now available for every visit.

#### 2.2.1 Participant consent

**FIGURE 1 alz70800-fig-0001:**
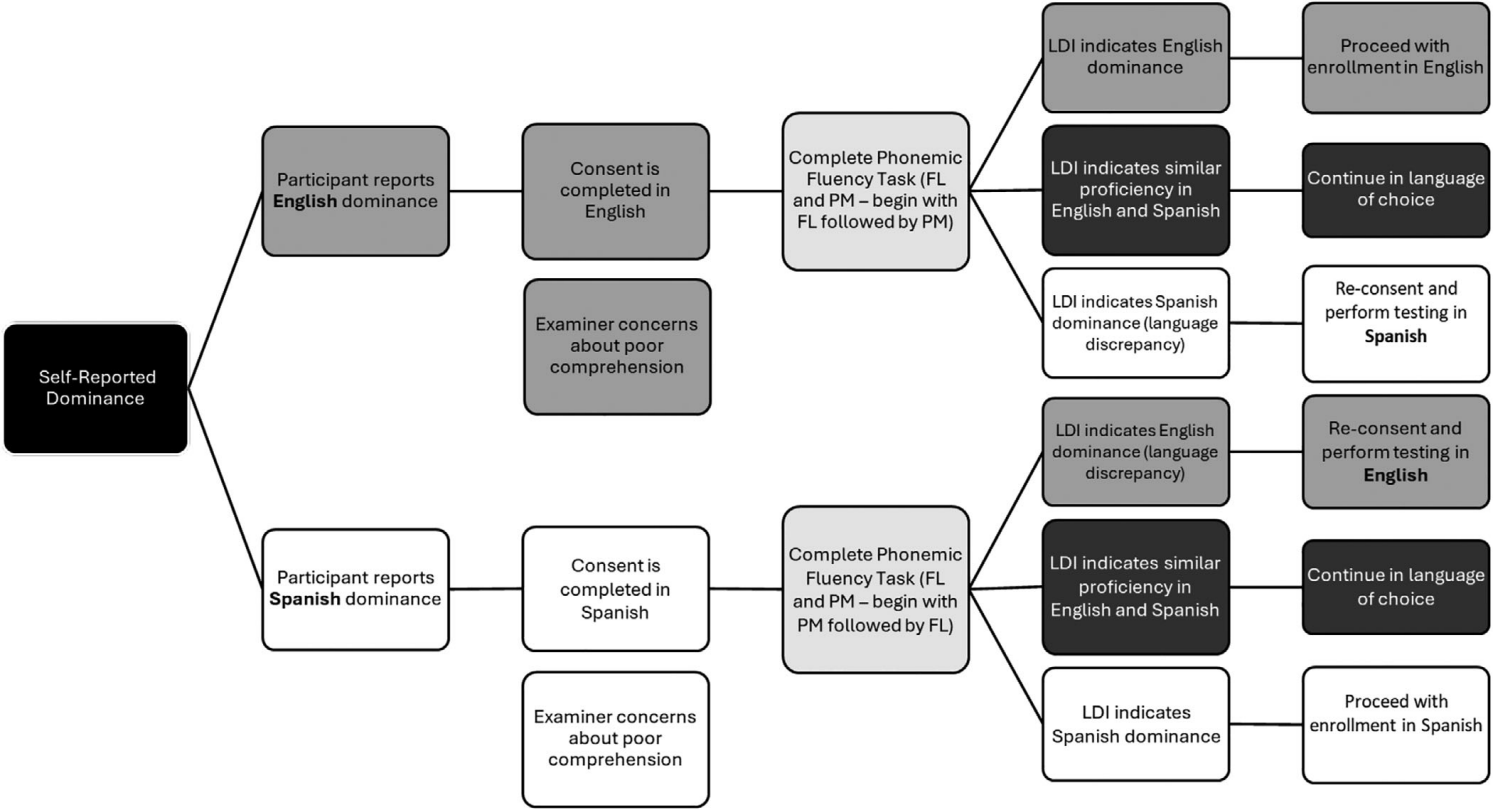
Language selection process. Process used to determine the language of evaluation for each participant. Decisions are based on LDI. LDI, language dominance index.

Starting with the initial recruitment, if a participant indicates that they are bilingual, they are asked to self‐identify their language preference (Figure 1). The consenting process continues in the identified preferred language. Following consent, all bilingual participants are administered the verbal fluency task in both English and Spanish to calculate LDI (see below for details). If the LDI differs from the participant's self‐reported language preference, the participant and research staff discuss the discrepancy from a script derived by neuropsychologists with clinical expertise and review the implications of completing the evaluation in their preferred language (see Section 3.1). For those individuals whose LDI scores indicate similar proficiency in English and Spanish, the study visit is continued in their language of choice. If the language discrepancy is large or there are concerns about poor comprehension, participants are re‐consented in the language indicated to be dominant by the LDI score. Thus, the language of the consent form should be consistent with that of the UDS assessment.

**RESEARCH IN CONTEXT**

1. **Systematic review**: The authors reviewed existing approaches to neuropsychological assessment of bilingual individuals, with an emphasis on measuring language proficiency. Prior studies highlight the need for more structured approaches to language assessment; however, standardized protocols to guide consistent implementation in bilingual evaluations are limited.
2. **Interpretation**: We applied a process to assess language proficiency in bilingual participants. We found that language influences cognitive test outcomes. Discrepancies between self‐reported and objectively measured language proficiency emphasize the complexities of evaluating bilingual individuals.
3. **Future directions**: Future research should focus on refining bilingual assessment protocols, establishing standardized guidelines, and exploring how language switching influences cognitive outcomes.

#### 2.2.2 Caregiver consent

**FIGURE 2:**
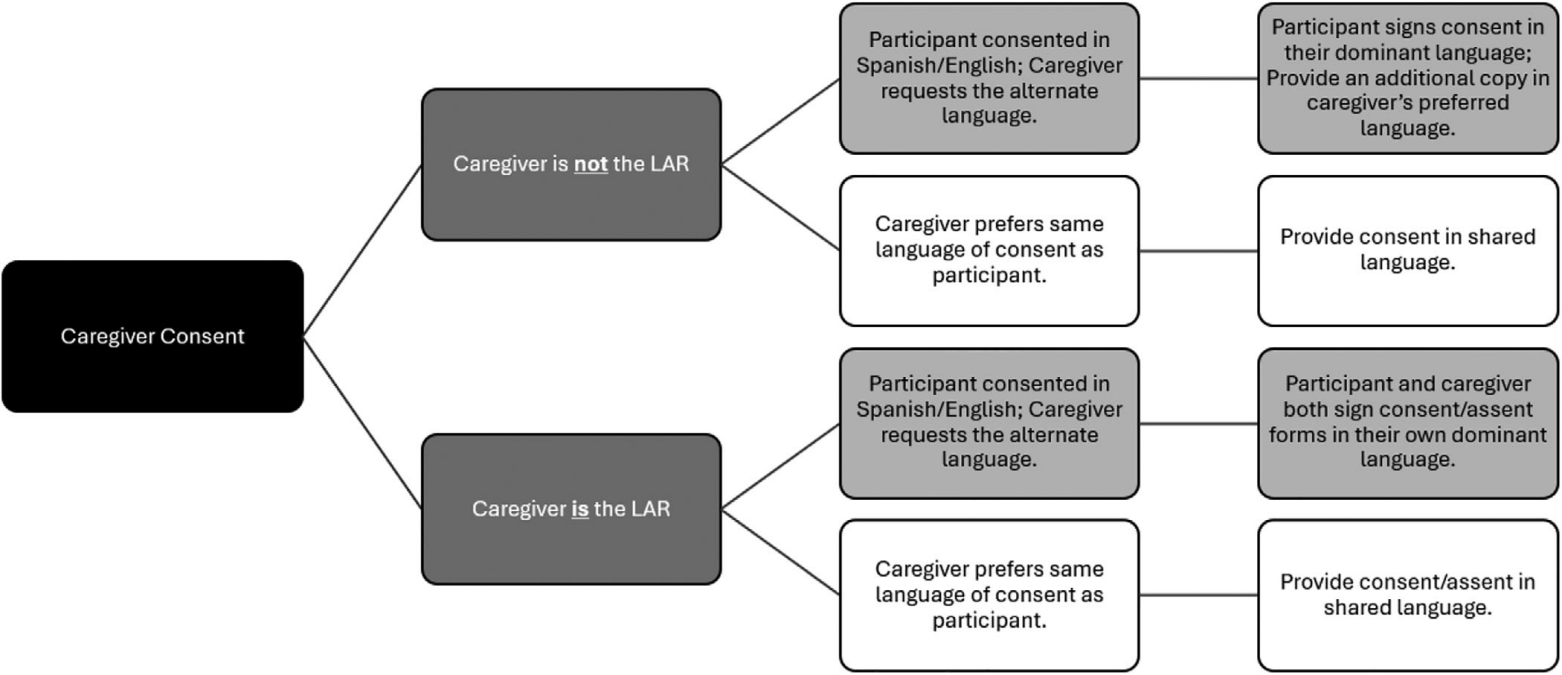
Approach for managing participant and caregiver language differences. Procedure for managing language differences between participants and their caregivers or LARs during the consent process. Caregivers are consented in their preferred language. If the participant's dominant language differs, an additional copy of the consent form is provided in the participant's language. LARs, legally authorized representatives.

Determining the language preference of the caregiver is done by directly asking them about their preferred language for communication.[Table alz70800-tbl-0001], [Fig alz70800-fig-0001] Often there are language differences within the same family. For example, the older participant may identify Spanish as their stronger language, whereas the younger caregiver identifies English as theirs. When this happens, we first identify whether the caregiver is the legally authorized representative (LAR) and follow the steps outlined in Figure 2 based on our experiences. This approach ensures that participants fully comprehend the study's purpose and expectations, and their rights, including the option to withdraw at any time. In addition, accommodating caregivers who prefer a different language guarantees they receive information in their preferred language without compromising the participant's understanding of the study.

#### 2.2.3 Interviewing family

The language used in collecting UDS forms for information such as family and medical history, is adjusted according to the caregiver's language preference and the participant's ability to contribute.

### 2.3 Measuring language

Participants who identify as bilingual are informed about the assessment process, which includes completing a form that collects information about the participant's language background and a verbal fluency task in both English and Spanish.

Implementation of the language of testing procedure proceeds in sequential steps (see Figure 1): (1) document self‐reported language preference and complete the CLS: Linguistic History Form; (2) administer verbal fluency tasks in both English and Spanish and obtain the LDI; (3) reconcile any mismatch (self‐report ≠ LDI) through a brief discussion with the participant, with LDI taking precedence (an LDI of 0.5 is treated as balanced and the participant selects the testing language); and (4) select the testing language and matching norms.

#### 2.3.1 CLS: Linguistic History Form

All bilingual participants complete the CLS: Linguistic History Form. Although we are collecting this information, it is not currently used to decide testing language. This form gathers information on linguistic background, such as the number of years spent in English‐ and Spanish‐speaking environments, the percentage of daily use of each language, and self‐reported proficiency in speaking, reading, writing, and understanding both languages using a scale from 1 (Almost None) to 7 (Like Native Speaker). We are determining self‐reported language proficiency by calculating an “average proficiency score” for English and Spanish, following the approach outlined by Marquine and colleagues.^43^ This score is derived by first averaging participant's self‐reported proficiency for Spanish and English and then dividing the Spanish average by the sum of both averages. Scores ≤0.49 were classified as Spanish dominant, scores = 0.5 were classified as balanced bilingual, and scores ≥0.51 were classified as English dominant.

#### 2.3.2 LDI

Participants complete a phonemic fluency task in both English and Spanish. This approach allows us to calculate their language dominance. We explain to participants that although they may be bilingual, one language often emerges as slightly more dominant, and identifying this helps us tailor services to better meet their needs and interpret their results more accurately. Language dominance is assessed objectively using the LDI, as outlined by Suarez et al.,^44^ through a phonemic fluency task in both languages to calculate a ratio. At the STAC, participants complete the UDS phonemic fluency task, which requires generating words that begin with “F” and “L” in English and “P” and “M” in Spanish. In our assessment process, the letters “F” and “L” are always used for generating words in English, whereas “P” and “M” are always used for generating words in Spanish. If a participant demonstrates stronger performance in Spanish—indicated by a lower LDI score (<0.5)—they are evaluated in Spanish by a Spanish‐speaking examiner. Conversely, if a participant shows stronger English performance, reflected in a higher LDI score, they are evaluated in English by an English‐speaking examiner, with support from a bilingual examiner when needed. We classified participants with a dominance index ≤0.49 as Spanish dominant, ≥0.51 as English dominant, and at 0.5 as balanced bilingual because it represents the intuitive midpoint of our LDI scale. Suarez et al.^44^ calculated the LDI from raw letter fluency counts and used data‐driven tertile splits (≤0.33, 0.34–0.66, >0.66) to classify participants as Spanish dominant, bilingual, or English dominant. Their cohort showed wide educational dispersion (mean = 12.5 years), whereas our STAC sample is more educated (mean = 15 years), producing a different proficiency distribution. Because those thresholds were specific to their cohort, and when applied to our sample, would have labeled 95% of participants as “balanced bilingual,” we instead adopted the 0.5 midpoint to distinguish Spanish versus English dominance. In our protocol, only participants who score exactly 0.5 on the LDI are considered “balanced bilingual.” For these individuals, we defer to their self‐reported language preference. Immediately after calculating the LDI, the examiner asks, “Which language do you feel most comfortable using for the rest of today's testing?” The evaluation is then performed in the chosen language. Future efforts will focus on establishing new thresholds to evaluate which best captures bilingual proficiency in our sample.

## 3 RESULTS

### 3.1 Baseline participant data

Table 1 shows differences in language exposure and self‐rated proficiency between participants tested first in English versus those tested first in Spanish. Participants tested in English reported more years spent in English‐speaking environments (mean ± SD: 65.4 ± 20.6 vs 52.0 ± 21.0), greater daily use of English (72.7% ± 25.1 vs 38.7% ± 31.7), and higher self‐rated English proficiency (6.5 ± 0.6 vs 5.8 ± 1.2) than those tested first in Spanish. In contrast, participants tested in Spanish showed greater exposure to Spanish (22.0 ± 27.9 vs 6.8 ± 15.8), more daily Spanish use (61.3% ± 31.7 vs 27.3% ± 25.1), and higher self‐rated Spanish proficiency (6.1 ± 1.0 vs 4.9 ± 1.3). These subjective patterns correspond with objective LDI scores, with the English‐testing group averaging 0.58 ± 0.07 (English dominant) and the Spanish‐testing group averaging 0.45 ± 0.07 (Spanish dominant).

### 3.2 Important considerations and common challenges

#### 3.2.1 LDI discrepancies

At the STAC, we have occasionally encountered situations where participants claim dominance in one language but demonstrate greater proficiency in another. The language environment shaped by the dominant culture plays an important role in how bilingual individuals assess their own language dominance.^47^ For example, within bilingual communities, English holds a privileged social position because of its association with formal and high‐status contexts^48^; consequently, English/Spanish bilinguals may overestimate their English proficiency and underestimate their Spanish proficiency, even when objective testing shows equal or stronger proficiency.^49^ The cultural pressure to conform to English‐speaking norms should be considered, which may influence self‐ratings, especially if individuals feel the need to present themselves as more proficient in English due to social or professional expectations.^50^ Based on our experience, we have found that psychoeducation is crucial in handling this scenario. Many participants may not understand why testing is conducted in one language versus another. In these situations, our examiners explain the importance of accuracy in testing and the provision of the best possible services (Refer to supplemental material for a suggested script). Examiners need to consider several factors before providing education. They should approach these topics with care to ensure that participants understand the context of the evaluation and feel supported in providing accurate information without feeling judged or misunderstood. Lack of trust in research procedures is common among Black and Hispanic/Latine communities.^51^ This distrust may arise from historical mistreatment or perceived biases against people of color.^3^, ^52^ To address this, examiners at our ADRC are encouraged to explain the research process clearly and simply, helping participants understand why we need to collect certain information and why it should be in their stronger language. Showing empathy while discussing these reasons can foster trust and improve participants’ understanding and comfort with the process.^53^ When LDI classification and stated preference differed, the examiner reviewed the discrepancy using neutral language (e.g., “It's common for what we expect about our stronger language to differ a little from test performance. These first tasks suggest you may show your abilities best in Spanish, so we'll continue in Spanish for the most accurate results. Please let me know if you have any concerns.”). In rare instances, participants have insisted on being tested in their non‐dominant language. In these cases, in an effort to maintain rapport, we acquiesce but document the decision for consensus/interpretation purposes. Due to staffing, scheduling constraints, participant burden, and potential practice effects, STAC opted to limit testing to the preferred language versus requesting testing in both languages as seen in some clinical practices. In our preliminary cohort among participants classified as balanced bilingual (*n* = 4), three preferred English. Ten participants whose LDI indicated Spanish dominance preferred English testing, and two whose LDI indicated English dominance preferred Spanish; all had at least 12 years of formal education. Given the small number of discrepant cases, analysis of demographic or linguistic correlates was not feasible.

#### 3.2.2 Language switching

As mentioned previously, bilingual individuals occasionally respond in both languages during the assessment. Allowing responses in both languages during testing helps with obtaining the most accurate information; however, these responses are difficult to score and do not adhere to standard test administration. During cognitive testing, participants are encouraged to use their dominant language as determined by the LDI. If a participant does not know a response in this language, they are encouraged to provide it in the other language, and a note will be made, but it will not be scored as accurate. Consistency in research methodologies is critical to maintaining reliability and validity across studies. Although clinical approaches that test the limits, such as allowing extensive switching between languages, may provide additional insights, these methods can introduce variability and reduce comparability across research settings. To evaluate whether the language of test administration influences performance, we are currently collecting qualitative data during the administration of a naming task (Multilingual Naming Test) by asking participants when they miss an item or are uncertain of an answer if they know it in the alternative language.

#### 3.2.3 Clinical interpretation

After completing the research visit, participant results are reviewed in a case consensus meeting. All clinical diagnoses at the ADRC are determined through a multidisciplinary consensus process. This process includes board‐certified behavioral neurologists, clinical neuropsychologists and postdoctoral fellows, research coordinators, and, when relevant, genetic counselors and/or geriatric psychiatrists. All members of the team are well versed in cultural awareness and many are bilingual. Consensus meetings occur weekly and are used to integrate findings across disciplines to arrive at a research‐level diagnosis consistent across sites (San Antonio, UT Rio Grande Valley, and Laredo). During consensus, available sociocultural information (e.g., country of origin, language of schooling, migration history) is discussed so that test findings are evaluated within an appropriate cultural‐linguistic context.

The team reviews the UDS for each participant, which includes detailed medical and psychiatric history, neurological and physical examination findings, neuropsychological testing results, informant‐based clinical interviews, and when available, structural neuroimaging. In cases where fluorodeoxyglucose–positron emission tomography (FDG‐PET) or cerebrospinal fluid (CSF) biomarkers are available, these are also considered.

For bilingual or Spanish‐speaking participants, particular attention is paid to testing language, language dominance (as determined by self‐report and/or verbal fluency ratio), and appropriateness of normative data. The neuropsychologist presents an interpretation of the participant's cognitive profile in the context of language use, educational background, cultural factors, and sociolinguistic context. The consensus team discusses how language preference and testing language may have influenced performance, and any diagnostic decisions are made in a culturally and linguistically informed framework based on clinical expertise. When diagnostic uncertainty exists, longitudinal follow‐up and additional biomarker data are often used to refine or revise diagnoses over time. The consensus process emphasizes diagnostic precision while also accounting for the sociocultural and linguistic diversity of the South Texas population.

A significant challenge in neuropsychological assessments of bilingual individuals is the scarcity of comprehensive normative data for this population, which can hinder the generalizability and interpretation of results. In response to this challenge, Marquine and colleagues^43^ published demographically‐adjusted normative data for version 3 of the UDS, specifically tailored for Spanish‐ and English‐speaking Hispanic/Latine individuals. These customized normative datasets enhance our ability to make precise interpretations of participants' results. During the presentation of neuropsychological findings, the most suitable normative reference group is carefully selected for each participant, guided by the following criteria: For Hispanic/Latine bilingual individuals, the LDI is calculated to guide language of assessment. Those with an LDI <0.5 are evaluated in Spanish using norms established by Marquine and colleagues,^43^ whereas those with an LDI >0.5 are evaluated in English using the same normative data. Non‐Hispanic/Latine participants are assessed in English using normative data from either Sachs and colleagues^54^ or Weintraub and colleagues.^55^ This process will likely change with the transition to version 4 of the UDS.

#### 3.2.4 Case examples

**FIGURE 3:**
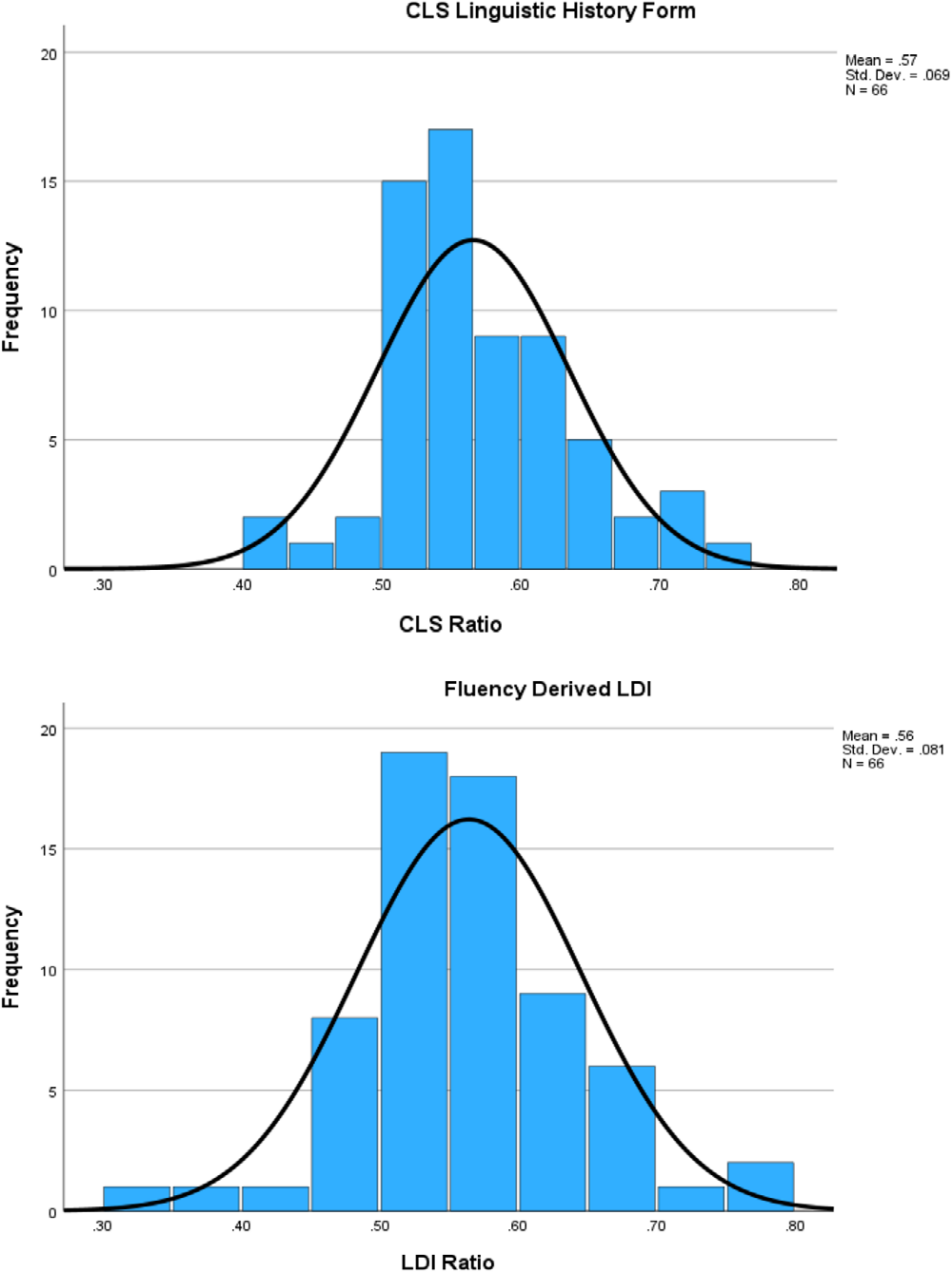
CLS: Linguistic History Form scores and fluency derived LDI scores. Participant scores on the CLS and LDI measures. Mean scores were 0.57 (CLS) and 0.56 (LDI), reflecting a slight overall dominance in English. Individual scores ranged from 0.40 to 0.77 for CLS and 0.33 to 1.0 for LDI, with most participants scoring between 0.5 and 0.6, suggesting a high degree of balanced bilingualism with a slight trend toward English dominance. CLS, CLS: Language History Form; LDI, language dominance index.

To date, 66 STAC participants completed the CLS and LDI. CLS scores had a mean of 0.57 and LDI scores yielded a mean score of 0.56, indicating a very slight dominance toward English proficiency. Participant CLS scores ranged from 0.40 to around 0.77, and LDI scores ranged from 0.33 to 1.0, with most scores clustering between 0.5 and 0.6 (Figure 3), indicating a high rate of balanced bilingualism (i.e., roughly equivalent proficiency in English and Spanish) with a slight a trend toward English dominance. Below, we outline two unique cases where calculating LDI served as a useful tool for identifying language dominance. These examples demonstrate the practical benefits and limitations of using the LDI in the assessment process. Language and norms were selected according to the procedure described in Section 2.3.

##### 3.2.4.1 Case AB

###### 3.2.4.1.1 Background and Presentation

Ms. AB is a 78‐year‐old right‐handed White Hispanic/Latine female who was born in Mexico and is bilingual in English and Spanish. Ms. AB completed 13 years of formal education. These details were considered during consensus to ensure that her test performance was interpreted within the context of her Mexican heritage. She reported a gradual decline in memory since age 72. Medical history was significant for type 2 diabetes, hypercholesterolemia, hypothyroidism, vitamin B12 deficiency, osteoarthritis, urinary incontinence, and sleep apnea (untreated). She denied any neurological or psychiatric symptoms. She worked until voluntary retirement at the age of 73. Family history is significant for Alzheimer's disease in both parents, with symptom onset in the mid‐ to late‐70s. Both parents passed away in their 80s. Ms. AB has several living siblings, all of whom are reportedly healthy without any known neurological or psychiatric diagnoses.

###### 3.2.4.1.2 Visit 1

**FIGURE 4:**
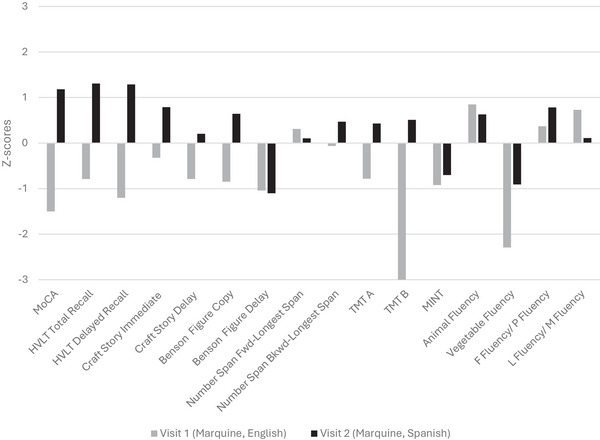
Comparison of neuropsychological scores: Visit 1 (English) versus Visit 2 (Spanish) for Ms. AB. Illustration of Ms. AB's neuropsychological test performance across two visits—with Visit 1 conducted in English and Visit 2 in Spanish—using normative data from Marquine et al. (2023) for the UDS‐3 battery. The graph compares scores across both sessions to highlight variability in test performance. UDS‐3, Uniform Data Set version 3.

Ms. AB was tested in English during her first visit (2022) based on her self‐reported preference. Her results were interpreted using the norms provided by Sachs and colleagues,^54^ as updated Marquine et al. norms^43^ were not yet available. The LDI was added to our testing protocol in 2023, and consequently, no LDI was obtained at Ms. AB's baseline visit. Ms. AB's neuropsychological tests during her first visit showed deficits in executive functioning, naming, and category fluency (vegetables) and low average performances on verbal and non‐verbal memory, simple visuomotor sequencing, and copy of a design. She was entirely independent for all aspects of daily living. Her research diagnosis was non‐amnestic mild cognitive impairment, multi‐domain subtype. After Marquine et al. norms^43^ were released in 2023, we re‐scored the 2022 raw data using the English norms for Hispanic/Latine individuals, generated z‐scores, and compared them with Ms. AB's results from her following visit in 2024 (see Figure 4). Under these norms, Ms. AB performed in the low average range on tasks measuring verbal and visual memory, design copy, simple visuomotor sequencing, and naming, whereas complex visuomotor sequencing and vegetable fluency were impaired. Even with cohort‐specific English norms applied, she would still meet criteria for non‐amnestic, multi‐domain MCI, suggesting that her baseline diagnosis was driven by genuine deficits in executive function or language of testing rather than by the choice of normative reference.

###### 3.2.4.1.3 Visit 2

During her second visit (2024), Ms. AB denied any interval changes in her cognitive abilities. Neurological and physical examination revealed a slow and unsteady gait attributed to hip pain. She reported independence in most activities of daily living. We had since implemented our language dominance assessment protocol, and her LDI was measured as 0.5, indicating equal dominance in Spanish and English. She was asked to select the language she used more frequently and with which she felt most comfortable, and she selected Spanish. The neuropsychological assessment was conducted in Spanish, this time using the Marquine and colleagues’ demographically‐adjusted norms^43^ for interpretation. She performed in the low average range in tasks assessing nonverbal memory and category fluency (vegetables) and her other performance across domains, including verbal memory, attention, executive functions, language, and visuospatial skills were within expectation for her age and education. Her neuropsychological profile no longer met criteria for MCI (see Table 2 for direct comparison). Of note, Ms. AB showed objective improvements in test performance that cannot be accounted for by normative differences (see Table 2 and Figure 4 using Marquine et al. norm corrections^43^ to facilitate direct comparisons). Is it unclear whether this rate of improvement is best attributed to testing in Spanish, familiarity with the testing procedures, normal intraindividual variability, or some combination thereof. However, we can confidently say that Ms. AB was doing well functionally, and that this most recent evaluation seems to reflect that more accurately. Ms. AB's letter fluency output was essentially stable across visits (Table 2). Spanish fluency was not collected at Visit 1 because the LDI had not yet been implemented. When Spanish fluency was performed at Visit 2, she produced a total of 28 Spanish words, identical to her Visit 2 English total, supporting our interpretation that her balanced performance at the second visit reflects equivalent proficiency.

**TABLE 2:** Raw score comparison of neuropsychological assessment results for Ms. AB.

	Visit 1 (2022; English) Raw scores	Visit 2 (2024; Spanish) Raw scores
MoCA	19	26
HVLT Total Recall (Trials 1–3)	19 (5, 7, 7)	25 (6, 11, 8)
HVLT Delayed Recall	5	9
HVLT Recognition Hits (false positives)	11 (2)	12 (3)
Craft Story 21 Immediate Verbatim	17	21
Craft Story 21 Delay Verbatim	12	15
Benson Complex Figure Copy	14	16
Benson Complex Figure Delay	7	7
Number Span Forward	6	5
Number Span Backward	4	4
Trail Making Test Part A	46”	47”
Trail Making Test Part B	300” (discontinued)	142”
MINT	25	26
Animal Fluency	22	20
Vegetable Fluency	6	9
F Fluency	14	13
L Fluency	14	15
P Fluency	–	16
M Fluency	–	12

*Note*: Ms. AB's raw scores on neuropsychological assessments from the UDS‐3 administered during two visits. Visit 1 conducted in English and Visit 2 in Spanish. The table includes tests across cognitive domains such as memory, attention, language, and executive function to illustrate potential variability in performance. Raw scores are provided to demonstrate performance across both language administrations.

Abbreviations: HVLT, Hopkins Verbal Learning Test; MoCA, Montreal Cognitive Assessment; MINT, Multilingual Naming Test; UDS‐3, Uniform Data Set version 3.

During consensus, her Mexican heritage and bilingual experience guided norm selection and test interpretation. Culturally unfamiliar vocabulary or fluency items were treated cautiously, and any naming weaknesses were interpreted within the context of bilingual lexical access.

##### 3.2.4.2 Case CD

###### 3.2.4.2.1 Background and Presentation

Ms. CD is a 76‐year‐old, Puerto Rican woman, bilingual in English and Spanish. She completed primary and secondary education in Puerto Rico and immigrated to the United States in the 1970s, where she later completed her bachelor's degree. She grew up in a bilingual household and received her schooling in both English and Spanish. Ms. CD reported a 4‐year history of progressive forgetfulness with associated changes in daily independence, primarily with managing medications. This is in the setting of heart murmur, osteoarthritis, asthma, untreated sleep apnea with chronically fractured sleep, carpal tunnel syndrome, and mild anxiety. In addition, she had a history of remote traumatic brain injury, with an unclear duration of loss of consciousness, secondary to an accident. Brain magnetic resonance imaging (MRI) revealed moderate global volume loss and white matter changes.

###### 3.2.4.2.2 Visit 1

During her first visit (2021), Ms. CD was evaluated in English based on personal preference, and her performance was interpreted using Sachs et al. non‐Hispanic White norms.^54^ Ms. CD's neuropsychological testing revealed poor attention and working memory, slowed cognitive processing, and a slight discrepancy between category‐cued and letter‐cued verbal fluency, as she had more difficulty with the latter. Copy of a design was below average. Verbal list learning revealed a relatively flat learning curve over successive trials with poor recall following delay. The provision of recognition cues improved her performance to the expected ranges. Her performance on story learning and memory and visual memory were within expectation. Her naming performance was lower than expected, an outcome we attributed in part to linguistic factors (e.g., bilingual individuals may know a concept in one language but may exhibit slower or failed retrieval in their other language, particularly for low‐frequency or context‐specific items, resulting in nonresponses or time‐outs).^15^ Her research diagnosis was amnestic multidomain dementia, with suspected mixed etiology (suspected vascular and Alzheimer's pathology). To examine whether a different set of norms would change this interpretation, we re‐scored Visit 1 data using Marquine et al. English norms for Hispanic/Latine individuals.^43^ Naming performance increased from impaired to low average, but the main weaknesses seen using Sachs et al. norms^54^ remained below expectation. Although the choice of norms moderated some scores, it did not alter the overall picture of cognitive impairment.

###### 3.2.4.2.3 Visit 2

During her second visit (2023), Ms. CD completed standardized language assessment and her LDI was 0.52. She was evaluated in English again with Marquine et al. norms^43^ used to interpret her neuropsychological results. Ms. CD's results were largely stable, showing improved visual attention/processing speed and subtle declines in learning and recall for both verbal and visual information. Ms. CD's research diagnosis remained unchanged, although the noted worsening of memory scores lent evidence for a primary Alzheimer's disease etiology.

###### 3.2.4.2.4 Visit 3

**FIGURE 5 alz70800-fig-0005:**
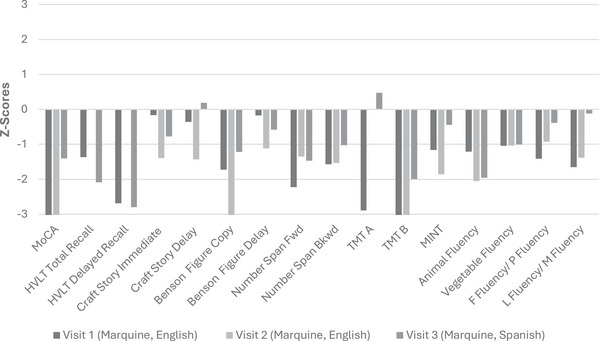
Comparison of neuropsychological scores: Visit 1 (English) versus Visit 2 (English) versus Visit 3 (Spanish) for Ms. CD. Illustration of Ms. CD's neuropsychological test performance across three visits—with Visit 1 and Visit 2 conducted in English and Visit 3 conducted in Spanish—using normative data from Marquine et al. (2023) for the UDS‐3 battery. The graph compares scores across three sessions to highlight variability in test performance. UDS‐3, Uniform Data Set version 3.

At this most recent visit (2024), Ms. CD opted to complete the testing in Spanish. Her LDI was measured as 0.33, which could represent normal testing variability or a shift in her language proficiency secondary to neurodegenerative disease/cognitive decline. Her testing was completed in Spanish based on her LDI. Once again, norms developed by Marquine and colleagues^43^ were used to interpret her neuropsychological evaluation results. Of interest, Ms. CD's performance showed improvements in the Montreal Cognitive Assessment (MoCA) and her naming was considerably stronger than either prior evaluation (see Table 3 and Figure 5). She continued to exhibit poor recall following delay, but similar to her initial evaluation, recognition cues proved helpful and she performed within expected ranges. Category fluency was variable, ranging from impaired (animals) to low average (vegetables). Her research diagnosis remained unchanged. Across the three evaluations, Ms. CD's letter fluency illustrated a shift from English to Spanish dominance (Table 3). In 2021, before the LDI was part of our protocol, she generated 14 English words. In her 2023 visit, her English total was 17 words and Spanish was 16 words, producing an LDI of 0.52. In 2024, her English output dropped to 12 words, whereas Spanish output increased to 24 words.

**TABLE 3:** Raw score comparison of neuropsychological assessment results for Ms. CD.

	Visit 1 (2021; English) Raw scores	Visit 2 (2023; English) Raw scores	Visit 3 (2024; Spanish) Raw scores
MoCA	14	14	18
HVLT Total Recall (Trials 1–3)	16 (4, 6, 6)	N/A	10 (1, 4, 5)
HVLT Delayed Recall	1	N/A	0
HVLT Recognition Hits (false positives)	10 (2)	N/A	10 (3)
Craft Story 21 Immediate Verbatim	20	12	13
Craft Story 21 Delay Verbatim	16	9	16
Benson Complex Figure Copy	13	11	13
Benson Complex Figure Delay	10	7	9
Number Span Forward	2	4	3
Number Span Backward	3	3	3
Trail Making Test Part A	69”	36”	39”
Trail Making Test Part B	256”	300” (discontinued)	300” (discontinued)
MINT	24	23	27
Animal Fluency	14	10	10
Vegetable Fluency	11	11	9
F Fluency	8	10	8
L Fluency	6	7	4
P Fluency	–	6	12
M Fluency	–	10	12

*Note*: Ms. CD's raw scores on the neuropsychological assessments from the UDS‐3, administered across three visits: Visit 1 and Visit 2 conducted in English, and Visit 3 in Spanish. The table includes tests across cognitive domains such as memory, attention, language, and executive function to illustrate potential variability in performance. Raw scores are provided to demonstrate performance across both language administrations.

Abbreviations: HVLT, Hopkins Verbal Learning Test; MoCA, Montreal Cognitive Assessment; MINT, Multilingual Naming Test; UDS‐3, Uniform Data Set version 3.

During consensus, we considered Ms. CD's reported schooling prior to immigration, her move to the United States in the 1970s, and completion of a bachelor's degree in the United States when selecting norms and interpreting her results. Lexical or fluency weaknesses were evaluated in the context of her bilingual exposure and cultural familiarity.

## 4 DISCUSSION

With the growing number of Hispanic/Latine individuals in the United States and the high incidence of dementia in this group, ADRCs should be prepared to conduct appropriate testing with English/Spanish bilingual individuals. Despite challenges in testing bilingual individuals, such as differences in language development, proficiency, and utilization, there are important considerations that can help increase validity of neuropsychological assessments and ensure appropriate services for this population.

Currently, there is no standardized protocol for neuropsychological testing of English/Spanish bilingual participants at ADRCs. In this article, we describe our current cohort of Hispanic/Latine participants and showcase the STAC's efforts to develop a more effective method for identifying language proficiency. We address the challenges we have encountered and share our lessons learned, with the goal of developing a structured procedure that facilitates consistent and reliable data collection and interpretation across ADRCs. We utilized the LDI^44^ as an objective tool for measuring language proficiency in bilingual participants. Baseline data from our 66 participants (Table 1) showed that those who tested in English reported more years in English‐speaking environments and higher daily English use, but also rated their English proficiency more highly, and these self‐reports were echoed by objectively higher LDI values. In contrast, participants who tested in Spanish showed the opposite pattern. These findings demonstrated that concordant subjective and objective measures may reinforce the validity of using more than one measure to assess language dominance for neuropsychological assessment. Of the four participants with an LDI of 0.50, three elected to be tested in English. Furthermore, 10 participants whose LDI indicated stronger Spanish performance opted for English testing, and two whose LDI indicated stronger English performance opted for Spanish. All of these individuals had at least 12 years of education, suggesting that educational background and related cultural or professional considerations may sometimes outweigh objective fluency scores. Although these cases are too few for formal subgroup comparisons, they demonstrate the importance of considering educational and contextual factors in addition to LDI and self‐report when determining the language of assessment.[Fig alz70800-fig-0005]

We present two case examples highlighting the impact of changes in LDI and the influence of language on cognitive assessment outcomes. In the first case (AB), the participant was assessed in English during her initial research visit and in Spanish during a follow‐up visit. Her case illustrates the significant influence that language selection and the use of appropriate normative data can have on assessment outcomes, as she was diagnosed with MCI during her first evaluation but demonstrated a normal cognitive exam during her second visit. Rescoring both visits with Marquine et al. norms^43^ demonstrated that the substantive factor driving her change from MCI to normal cognition was the change in test language (English vs Spanish), rather than differences in normative reference. This improvement may reflect reduced processing demands of using her dominant language, allowing more efficient word retrieval and decreased executive control load. The second case (CD) involves a participant who completed three research visits. She was tested in English during her first two visits, but due to a change in her LDI, she was assessed in Spanish during her third visit. Although her performance on most cognitive domains remained stable across visits, her naming ability showed significant improvement during her last visit. This reversal may be attributed to increased daily exposure to Spanish, test–retest variability, or advancing neurodegeneration.

Although our site is well‐positioned to develop culturally and linguistically responsive procedures given the strong Hispanic/Latine and bilingual population in South Texas, there remain inherent limitations in bilingual cognitive assessment, even when standardized protocols are followed. One major limitation is that we did not systematically collect critical sociodemographic details (e.g., age at second‐language acquisition, place/language of education, acculturation level), which can critically shape language dominance and guide the choice of testing language. UDS‐4 now includes these measures, and we will leverage them in future analysis of a larger sample to refine our assessment. Although the use of an objective metric such as the LDI can help identify the dominant language for testing, the LDI is not a direct measure of overall language proficiency. Instead, it offers a practical and informative estimate of expressive language use, particularly when verbal fluency tasks are administered in both languages. Miranda et al.^41^ demonstrated that LDI scores were significantly associated with neuropsychological test performance in bilingual adults and were more predictive of performance than comprehensive language batteries, supporting the utility of verbal fluency as a proxy for language dominance in applied settings.

Although recent guidance (e.g., Arce Rentería et al.^56^) recommends using multidimensional tools to assess language experience and proficiency, such tools were not collected systematically across our cohort. Therefore, verbal fluency was selected as the most feasible and empirically supported alternative, allowing for consistent assessment across participants. We recognize that self‐report and interview‐based tools provide a more nuanced understanding of language use, but in our study, methodological consistency and data availability guided our decision. Some participants who were tested in Spanish responded regularly in English, requiring repeated reminders to stay within the testing language. These patterns underscore the fluid nature of bilingualism and the challenges it presents for fixed‐language testing protocols. As a next step, we plan to conduct brief interviews with participants whose self‐reported language preference is different from their LDI score to document the reasons for these discrepancies. Future work should focus on incorporating more flexible approaches to language use during testing and developing norms that reflect the complex linguistic realities of bilingual individuals.

Although the LDI currently takes precedence when it conflicts with self‐report, this practice is provisional. Given the limitations of relying on the LDI, and as our cohort grows, we are currently refining our evaluation process at the STAC by collecting language information from multiple sources, with the goal of identifying the most effective method for assessing language dominance. First, we are gathering the participants’ self‐reported language preference. Second, we are administering the UDS phonemic fluency tasks in English (letters F and L) and Spanish (letters P and M) to compute the LDI. Third, we are administering the CLS: Linguistic History Form and determining self‐reported language proficiency by calculating an “average proficiency score” for English and Spanish, as outlined by Marquine and colleagues.^43^ We are actively working on comparing self‐reported language proficiency scores from the CLS: Linguistic History Form with the objective LDI to determine the level of agreement between these two methods in establishing language dominance. Finally, we are collecting qualitative data from the Multilingual Naming Test to assess whether participants can access their lexical knowledge in the alternative language by documenting when participants correctly answer items in their alternative language after missing them in their primary testing language. We aim to assess if the test language (as determined by the LDI) is related to the participant's performance on the Multilingual Naming Test. This comprehensive approach lays the groundwork for developing a standardized procedure to determine language dominance, facilitating a more accurate test‐language selection.

Reaching a consensus on practical guidelines for determining the language of testing is paramount and essential for advancing the field of neuropsychology to effectively assess linguistic and cultural minority individuals. Given the multi‐site nature of the NACC database, it is important to standardize the assessment of language proficiency to ensure comparability of data. With the introduction of the UDS‐4, we need to adapt our approach to better serve our population. We must incorporate culturally relevant assessments and more effectively address health disparities to enhance participant outcomes. Although in this article we aim to describe and document the STAC's procedures, it does not attempt to establish or propose formal consensus guidelines across sites. We recognize the urgent need for greater harmonization, and we hope that detailed descriptions of site‐level practices, such as the one presented here, can serve as a starting point for identifying shared practices, challenges, and areas of divergence. We would welcome future efforts to engage ADRC sites in structured discussions aimed at developing best practice guidelines for consensus diagnosis, especially in linguistically and culturally diverse populations.

## CONSENT STATEMENT

Written informed consent was obtained from each National Alzheimer's Coordinating Center (NACC) participant or their legally authorized representative.

## CONFLICT OF INTEREST STATEMENT

The authors declare no conflicts of interest. Any author disclosures are available in the supporting information.

## Supporting information



Supporting Information

Supporting Information
