# Supplementary material for: Addressing language challenges in bilingual neuropsychological assessments at the South Texas Alzheimer's Disease Research Center (ADRC)
Source: Alzheimers Dement. 2025 Oct 16;21(10):e70800. doi: 10.1002/alz.70800 (PMC12531422; doi:10.1002/alz.70800)
Supplement: Supplementary file 1 — Supporting Information [file ALZ-21-e70800-s002.docx]

Supplemental Material

Sample Script for Introducing the Neuropsychological Evaluation and Language Dominance Index (English/Spanish)

**Introducing Neuropsychological Testing**

English:

*“Thank you for your interest in participating in our research study. As part of the testing process, we will be doing a neuropsychological evaluation. During this evaluation, you will be asked to complete some tasks that measure different aspects of your thinking and behavior. This evaluation will help us better understand how your brain is working and can help identify strengths and areas where you might be having more difficulties. We just ask that you try you very best.”*

Spanish:

*“Gracias por su interés en participar en nuestro estudio de investigación. Como parte del proceso de evaluación, realizaremos una evaluación neuropsicológica. Durante la evaluación, se le pedirá que complete algunas tareas que miden diferentes aspectos de su pensamiento y comportamiento. Esta evaluación nos ayudará a comprender mejor como está funcionando su cerebro y puede ayudarnos a identificar tanto sus fortalezas como las áreas donde podría estar experimentando más dificultades. Solo le pedimos que haga su mayor esfuerzo.”*

**Introducing the Language Dominance Index**

English:

*“We will begin with a verbal task in both English and Spanish. Many bilingual individuals are more fluent or express themselves more easily in one language than the other, even if they use both regularly. This task will helps us choose the language that will allow you to perform at your best during testing. With this information, we can interpret your results more accurately and provide an evaluation that is fair and tailored to your needs.”*

Spanish:

*“Comenzaremos con una prueba/tarea verbal en inglés y en español. Muchas personas bilingües tienen mayor fluidez o se expresan con más facilidad en un idioma que en otro, incluso si usan ambos con regularidad. Esta prueba/tarea nos ayuda a identificar el idioma que le permitirá desempeñarse lo mejor posible durante la evaluación. Con esta información, podremos interpretar sus resultados con mayor precisión y ofrecerle una evaluación justa y adaptada a sus necesidades.”*
